# RETRACTED: Jing et al. Shear Strengthening of Deep T-Section RC Beams with CFRP Bars. *Materials* 2021, *14*, 6103

**DOI:** 10.3390/ma19091902

**Published:** 2026-05-06

**Authors:** Zheng-Nan Jing, Rong-Gui Liu, Gui-Hua Xie, Dan Liu

**Affiliations:** 1 Faculty of Civil Engineering and Mechanics, Jiangsu University, Zhenjiang 212013, China; jingzhengnan@sina.com (Z.-N.J.); liurg@ujs.edu.cn (R.-G.L.); 2 Architectural Engineering Institute, Jinling Institute of Technology, Nanjing 211169, China; liudan@jit.edu.cn

The journal retracts the article titled “Shear Strengthening of Deep T-Section RC Beams with CFRP Bars” [1], cited above.

Following publication, the authors contacted the Editorial Office regarding an overlap between this article [1] and an earlier publication [2] originating from a similar authorship group and published in another language.

Adhering to our standard procedure, the Editorial Office and Editorial Board conducted an investigation that confirmed a significant overlap between two publications [1,2] without appropriate citation and arranging appropriate copyright permission. As a result, the Editorial Board have decided to retract this paper as per MDPI’s retraction policy (https://www.mdpi.com/ethics#_bookmark30, accessed on 26 March 2026).

This retraction was approved by the Editor-in-Chief of the journal *Materials*.

The authors did not provide a comment on this decision.
